# A Unique Case of the Syndrome of Irreversible Lithium-Effectuated Neurotoxicity (SILENT) Presenting With Multiple Neurological Sequelae

**DOI:** 10.7759/cureus.38102

**Published:** 2023-04-25

**Authors:** Abdelhadi Farouji, Arwa Battah, Amaar S Ahmad, Iyad Farouji, Richard Miller

**Affiliations:** 1 Department of Internal Medicine, Saint Michael's Medical Centre, New York Medical College, Newark, USA; 2 Department of Pulmonary and Critical Care Medicine, Saint Michael's Medical Centre, New York Medical College, Newark, USA

**Keywords:** ataxia, expressive aphasia, irreversible neurological sequalae, lithium toxicity, silent syndrome

## Abstract

Lithium can have toxic effects on the central nervous system (CNS) that can be both acute and chronic. The syndrome of irreversible lithium-effectuated neurotoxicity (SILENT) was suggested in the 1980s to describe lithium intoxication-induced persistent neurological sequelae. In this article, we report a 61-year-old patient with bipolar disorder who had developed expressive aphasia, ataxia, cogwheel rigidity, and fine tremors after acute on chronic lithium toxicity. These neurological symptoms remained for four months after discontinuation of lithium, confirming the persistence of CNS signs and symptoms, which makes this case meets the SILENT syndrome criteria. Although rare, our report - which shows a severe and disabling form of SILENT syndrome - highlights the need for additional caution when treating patients with lithium and the need to perform strict control of the putative risk factors argued to be associated with the development of this syndrome.

## Introduction

Lithium is an effective mood stabilizer that is used principally for the management of bipolar disorder (BD) [1]. Since it has a narrow therapeutic index, lithium toxicity and severe side effects have been frequently reported in literature [2]. Patients experiencing lithium toxicity typically present with neurological findings, including ataxia, tremor, confusion, agitation, gait disturbances, hyperreflexia, dysarthria, and muscle weakness. Severe lithium intoxication can lead to seizures, non-convulsive status epilepticus, and encephalopathy [3]. Fortunately, in most cases, full recovery follows when the lithium treatment is discontinued. However, in rare cases, lithium-induced persistent neurological sequelae have been reported [4]. Herein, we are presenting a 61-year-old male patient who developed expressive aphasia, cogwheel rigidity, ataxia, and fine tremors secondary to lithium toxicity, which showed partial improvement; however, it did not resolve completely after four months.

## Case presentation

We present a 61-year-old male patient with a past medical history of bipolar disorder, coronary artery disease status post percutaneous coronary intervention with stent placement in 2001, hypertension, hyperlipidemia, and chronic obstructive pulmonary disease (COPD). He was diagnosed with bipolar disorder at the age of 46 and placed on lithium treatment two years prior to his presentation to our facility. His medications include lithium extended release 450 mg twice daily, mirtazapine 45 mg daily, quetiapine 300 mg daily, gabapentin 800 mg three times daily, doxepin 100 mg twice daily, and atorvastatin 40 mg daily. 

Due to multiple issues in his life causing the worsening of his symptoms, he decided to take six lithium pills at the same time to find relief. After this incident, the patient's family noticed that he became confused, disoriented, and had an unsteady gait, and because of these symptoms, they brought him to the emergency department.

During the initial encounter in the emergency department, he was hemodynamically stable but had a fever of 38.2 C. He was confused, disoriented, agitated, and his thoughts were incoherent. Additionally, the patient was found to be experiencing auditory hallucinations. Haloperidol was given, and restraints were placed. Laboratory test results revealed lithium toxicity, with a lithium level of more than 3.0 millimoles/liter (normal range: 0.60 - 1.20 millimoles/liter), creatinine level of 1.1 milligrams/deciliter (normal range: 0.60 - 1.20 milligrams/deciliter), and ethanol level of less than 0.30 milligrams/deciliter. An electrocardiogram showed a mild prolonged QTc-interval of 472 milliseconds (normal range <440 milliseconds in men). Other laboratory tests were within normal limits. Intravenous fluids were started, and he was transferred to the intensive care unit (ICU) for further evaluation and management.

The plan was to hold all his psychiatric medications, including mirtazapine, quetiapine, doxepin, and gabapentin; a double-lumen dialysis catheter was placed, and a four-hour hemodialysis session was done. His lithium level decreased six hours after hemodialysis to 1.53 millimoles/liter, and he began to be less agitated and more oriented; however, he remained mildly confused. On the third day of his hospital stay, his lithium level again decreased to 0.52 millimoles/liter. However, he was found to be nonverbal yet could follow simple commands. On neurological examination, his pupils were equal and reactive to light and accommodation, no afferent pupillary defect, normal extraocular eye movements, no facial asymmetry noted, he had increased neck and bilateral upper limb tone with cogwheel rigidity, bilateral fine resting hand tremors, ataxic gait, normal lower limb tone, motor power 5/5 in proximal and distal bilateral upper and lower extremities, normal and symmetric deep tendon reflexes, normal bilateral plantar reflexes.

**Figure 1:**
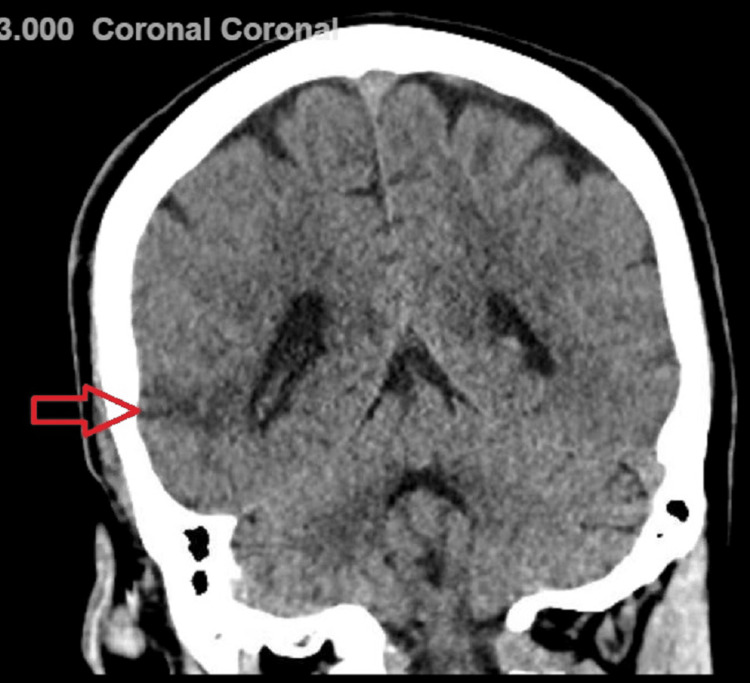
CT scan without contrast, coronal section, revealed indeterminate age infarct in the right temporal lobe (red arrow)

**Figure 2:**
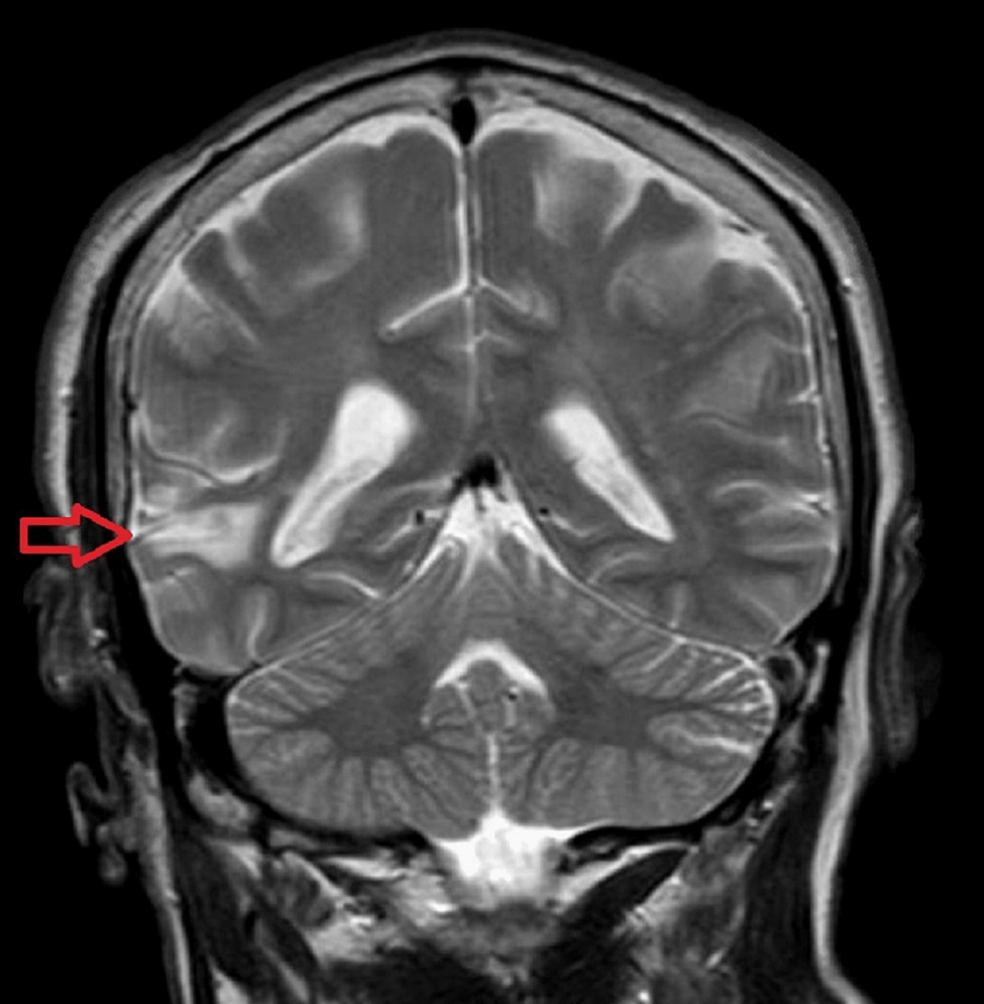
MRI, T2, coronal section, revealed encephalomalacia in the right temporal lobe (red arrow)

Head CT without contrast revealed low attenuation in the right temporal lobe with loss of gray matter/white matter differentiation concerning for infraction of an indeterminate age (Figure 1). Brain magnetic resonance imaging (MRI) revealed mild encephalomalacia in the right temporal lobe, corresponding to the CT finding, felt to be related to an old infarct and no enhancement in this region (Figure 2). There was prominence of the ventricular system and CSF spaces, suggesting mild cerebral atrophy. Two-hour-extended video electroencephalography (EEG) showed mild and diffuse slowing of background activity and increased slowing in the right hemisphere during sleep, without any epileptiform discharges. Vitamin B12 levels were within normal range, rapid plasma reagin (RPR) was non-reactive, and thyroid stimulating hormone (TSH) level was 1.174 uIU/mL (normal range: 0.5 - 5.0 uUI/mL). Four months later, with speech and physical therapy, he began to speak; however, his speech remained slow and low-pitched, and he continued to complain of mild neck and bilateral upper limb rigidity, bilateral fine hand tremors, and ataxic gait. Since these sequelae were persistent four months after lithium toxicity and could not be explained by other pathology, it raised the suspicion that he developed irreversible changes secondary to lithium neurotoxicity. This entity is known as the syndrome of irreversible lithium-effectuated neurotoxicity (SILENT).

## Discussion

Lithium has been widely used in the treatment of psychiatric and neurological disorders. It is the drug of choice for maintenance treatment for bipolar disorder and can be used in the treatment of mania, hypomania, and bipolar depression [1]. It is associated with a reduced risk of suicide attempts and suicidal deaths in bipolar patients [5]. The mechanism of action is unknown, but multiple studies suggest that it may decrease the excitability of the neurons in patients with bipolar disorders [6].

Lithium has a narrow therapeutic index. The target serum level for bipolar management is between 0.6 and 1.0 mmol/L, and toxicity can develop when the level reaches more than 1.5 mmol/L [7]. Because of this narrow therapeutic range, many patients may develop toxicity [2]. Lithium toxicity can be classified into three categories according to the chronicity of exposure (Table 1), with acute on chronic, as in our case, being the most severe form with the greatest risk of irreversible neurological consequences [8,9].

**Table 1:** Categories of lithium toxicity according to the chronicity of exposure Source: Hedya et al. [9]

Category	Definition	Symptoms
Acute	Acute overdose in a patient who does not take lithium regularly	Mainly GI and can progress to neurological
Chronic	Chronic over-medication or drug accumulation	GI and neurological
Acute on chronic	Acute overdose in a patient who takes lithium daily	Mainly neurological

In patients with acute toxicity and acute on chronic toxicity, neurologic findings develop later in the course of illness because time is required for the drug to be distributed within the central nervous system (CNS). High serum lithium levels can cause encephalopathy with an altered mental state, cerebellar dysfunction (dysarthria, ataxia, and nystagmus), seizures, and rigidity. Peripheral manifestations of lithium toxicity include myasthenia-like syndrome, proximal muscle weakness, and rhabdomyolysis [10,11]. Additionally, some of the neurological findings occur at therapeutic levels. These changes include tremors, cognitive changes, and lack of coordination, but are typically tolerable and not permanent. Therapeutic lithium tremor is generally symmetric, limited to the arms, and non-progressive. But the tremor from lithium toxicity is coarser and more disabling, may also affect the lower extremities, and occurs with other symptoms of toxicity [11,12].

Usually, the neurological symptoms are not persistent and disappear with the reduction of serum lithium levels. However, if these symptoms, such as cerebellar impairment, extrapyramidal symptoms, and dementia, persist for two months or more after drug cessation, toxicity is labeled irreversible. In 1987, Adityanjee et al. suggested the acronym SILENT to describe the lithium-induced neurological symptoms that persist for at least two months after the discontinuation of the drug in the absence of previous neurological impairment [4,13]. In this case, we described a 61- year-old male patient who developed expressive aphasia, ataxia, fine tremors, and extrapyramidal symptoms after an acute on chronic lithium toxicity; these symptoms improved with time but did not resolve completely.

The exact mechanism of persistent neurotoxicity is unclear until now. Extensive demyelination has been found on biopsy in the peripheral nerves involved in lithium-induced peripheral neuropathy [14]. This finding suggests that similar changes occur in the CNS [15]. Also, several mechanisms, such as cerebellar atrophy, loss of Purkinje cells, and gliosis of the cerebellar cortex, have been described as the cause of lithium-induced cerebellar dysfunction, but the mechanism of these remarkably selective pathologic changes is not well understood [16].

A direct correlation between serum levels and the development of neurological sequelae is questioned. Several cases of SILENT syndrome have been reported with normal lithium serum levels. This can be explained by the pharmacokinetics features of lithium. The permeability of the blood-brain barrier to lithium is reduced in comparison to other tissues of the body. Therefore, the distribution of lithium in the brain is delayed by approximately 24 hours as compared with that in the plasma. Since the therapeutic effects and lithium-induced neurological sequelae depend on the lithium level inside the brain, many patients with acute intoxication, such as suicidal attempts, may present with high serum levels without any neurological symptoms, at least initially. On the contrary, the brain lithium levels of patients with chronic lithium use have enough time to develop. Therefore, patients on chronic therapy are more susceptible to adverse neurological effects after an acute rise in lithium [17].

Fever, high serum levels during acute lithium intoxication, and concomitant use of antipsychotics or antidepressants are described as potential risk factors for developing lithium-induced neurological sequelae. Other precipitating factors include hypertension, chronic renal failure, heart failure, the rapid correction of hyponatremia or hyperlithemia, preexisting neurologic illness, and epilepsy [4]. Fever can be secondary to the intoxication itself, or it can be caused by secondary infection. The mechanism by which the fever increases the risk of lithium-induced neurological sequelae is not well understood. There is a hypothesis that fever increases the permeability of the blood-brain barrier that increases the brain lithium levels [16].

We describe a new case of a 61-year-old patient who developed expressive aphasia, ataxia, cogwheel rigidity, and fine tremors secondary to lithium toxicity that did not resolve completely after four months. This new case of SILENT syndrome may help us to know more about this uncommon entity; the unique part of this case is that the patient developed symptoms not limited to only one brain region but to different regions such as the cerebrum, basal ganglia, and the cerebellum. 

## Conclusions

Lithium can cause reversible neurological symptoms; however, in rare cases, permanent irreversible symptoms have been reported. High serum level or lithium toxicity is not always required to develop irreversible neurological sequelae and SILENT syndrome and can be developed with therapeutic serum levels. This case highlights the importance of following up with patients on lithium therapy to perform regular neurological exams, monitor serum lithium levels and renal function, and monitor patients for early signs of toxicity even when patients are within the therapeutic range of lithium. In this case, the presence of fever and the concomitant use of antipsychotics may contribute to the development of irreversible neurological sequelae like in other cases. There is an urgent need for additional studies to assist in the identification of the risk factors for SILENT syndrome and how it can be effectively treated in a timely manner.
